# Electrochemical Disinfection in Water and Wastewater Treatment: Identifying Impacts of Water Quality and Operating Conditions on Performance

**DOI:** 10.1021/acs.est.0c06254

**Published:** 2021-02-22

**Authors:** Steven Hand, Roland D. Cusick

**Affiliations:** Department of Civil and Environmental Engineering University of Illinois at Urbana−Champaign, Urbana, Illinois 61801-2352, United States

## Abstract

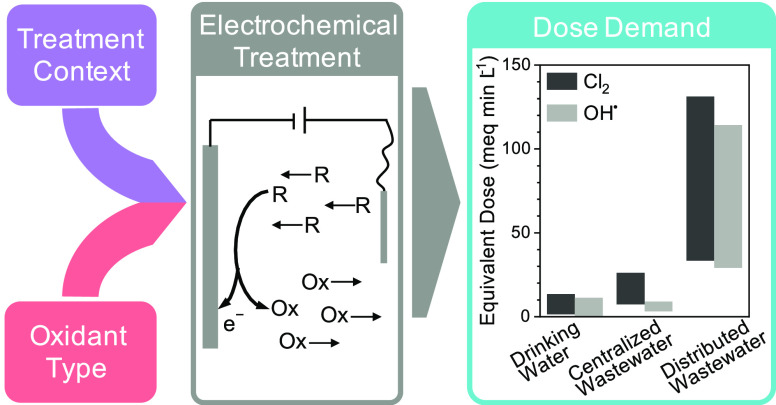

Electrochemical disinfection—a method in which chemical oxidants are generated *in situ* via redox reactions on the surface of an electrode—has attracted increased attention in recent years as an alternative to traditional chemical dosing disinfection methods. Because electrochemical disinfection does not entail the transport and storage of hazardous materials and can be scaled across centralized and distributed treatment contexts, it shows promise for use both in resource limited settings and as a supplement for aging centralized systems. In this Critical Review, we explore the significance of treatment context, oxidant selection, and operating practice on electrochemical disinfection system performance. We analyze the impacts of water composition on oxidant demand and required disinfectant dose across drinking water, centralized wastewater, and distributed wastewater treatment contexts for both free chlorine- and hydroxyl-radical-based systems. Drivers of energy consumption during oxidant generation are identified, and the energetic performance of experimentally reported electrochemical disinfection systems are evaluated against optimal modeled performance. We also highlight promising applications and operational strategies for electrochemical disinfection and propose reporting standards for future work.

## 1 Introduction

Waterborne pathogenic bacteria and viruses were responsible for an estimated 842,000 diarrhea related deaths due to insufficient water quality, sanitation, and hygiene in 2012.^1,2^ In combination with handwashing and other hygienic practices which reduce person-to-person pathogen transmission, water treatment reduces disease by decreasing pathogen transmission from the environment.^3−5^ A significant source of pathogens in water treatment are due to fecal contamination from inadequate or improperly managed sanitation systems.^6,7^ In these contexts, open defecation or improperly sited sanitation interventions such as pit latrines leach wastewater and associated pathogens into onsite and downstream water supplies.^8,9^ As of 2018 an estimated 4.5 billion people do not have access to safely managed sanitation services, almost 900 million of whom still practice open defecation.^10^ Within these resource limited settings, distributed treatment systems have been developed and studied as a tool to destroy wastewater pollutants including pathogens and reach the United Nations’ Sustainable Development Goal (SDG) of global access to equitable and adequate sanitation by 2030.^1,10−15^

In countries with widespread centralized water and wastewater treatment systems, pathogen inactivation levels are regulated by governing authorities to ensure water is safe for human consumption or discharge into receiving water bodies.^16,17^ In this paradigm, disinfection is typically carried out prior to potable water distribution or discharge of treated wastewater to receiving water bodies.^18−20^ However, aging centralized treatment infrastructure has contributed to unsafe levels of pathogen exposure, such as recent *Legionella* outbreaks in the U.S., leading to increasing interest in distributed disinfection systems, wherein pathogen inactivation occurs at the site of water consumption or wastewater generation.^21−23^ For most centralized water and wastewater treatment paradigms, pathogenic organisms are inactivated through the application of oxidants such as free chlorine, chlorine dioxide, chloramines, ozone, and ultraviolet (UV) radiation.^24−31^ These oxidants are supplied from external sources (e.g., Cl_2_, NaOCl, ClO_2_, O_3_, UV irradiation) as a final stage during water and wastewater treatment.^25,32,33^ Within resource limited settings, pathogen removal or inactivation has often been achieved through point-of-use water treatment technologies such as filters and combined coagulant/oxidant sachets.^34−36^ However, these technologies have varying pathogen treatment efficacies (e.g., 0.5−2 log reduction of viruses with point-of-use technologies vs 3 log reduction with free chlorine) as compared to more robust chemical oxidation systems such as chlorination, ozonation, and UV disinfection systems (all of which have been shown to achieve at least 3 log reduction of viruses)^33^ and therefore carry a greater risk of exposing users to unsafe pathogen levels.^36^

A promising approach to disinfection which could be leveraged in both centralized and distributed treatment contexts is electrochemical oxidation.^12,37−42^ In electrochemical disinfection, an oxidant is generated *in situ* via redox reactions on the surface of an electrode.^12,43−47^ Just as with traditional disinfection techniques, numerous electrochemically generated oxidants can be used in disinfection including Cl_2_,^37,40,47−49^ O_3_,^44,50,51^ SO_4_^•–^,^52,53^ and OH^•^.^54−56^ Due to modular cell design, electrochemical oxidation can potentially be scaled across centralized and distributed treatment contexts.^57−59^ Likewise, electrochemical oxidant generation can operated in resource limited settings that often lack transportation and electrical power distribution infrastructure by using electricity generated onsite through renewable energy sources such as photovoltaic cells with minimal external chemical additions.^42,60,61^ While there are an increasing number of studies investigating electrochemical oxidation/disinfection, there has been inconsistent reporting of disinfectant dose generation, energy consumption, and pathogen inactivation. Likewise, little focus has been devoted to contextualizing the potential challenges of applying electrochemical disinfection systems in both water and wastewater treatment.

This Critical Review explores the impacts of treatment context, oxidant selection, and operating practice on the reported and potential performance limits of electrochemical disinfection systems in terms of oxidant dose and electrical energy consumption. We first review chemical interactions between the chemical composition of water across treatment contexts, the characteristics of common electrochemically generated oxidants, and how water composition and oxidant selection influence disinfectant requirements. We then survey the relationship between dose generation and energy consumption, with a detailed analysis of the operating conditions which serve as primary drivers of energy consumption. Last, we propose reporting standards for future electrochemical disinfection studies and recommend pathways for future development.

## 2 Electrochemical Disinfection

**Figure 1:**
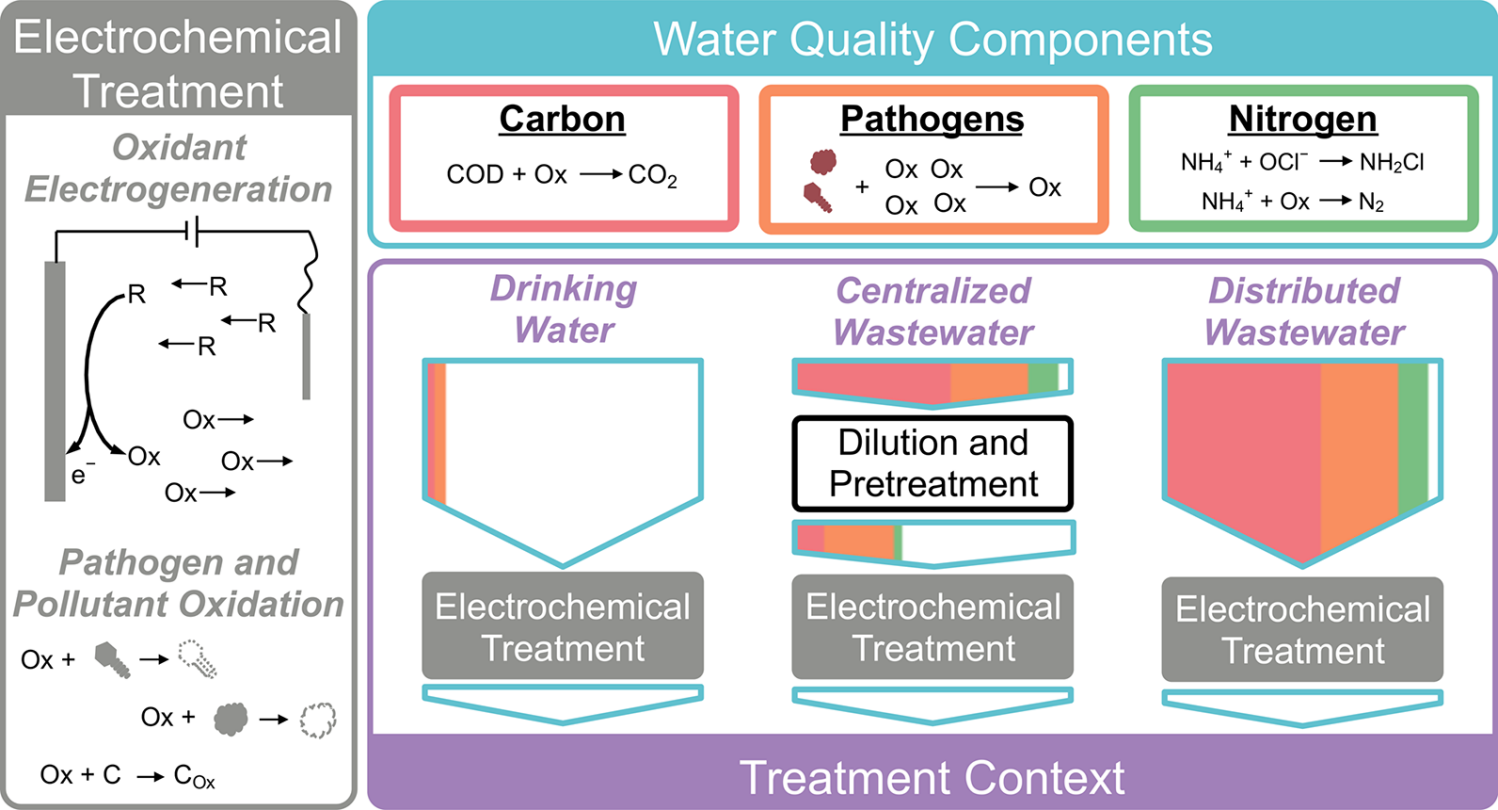
Electrochemical disinfection generates oxidants *in situ* to deactivate pathogens and chemically transform reduced carbon and nitrogen molecules. Differences in water quality (most importantly carbonaceous COD, ammonia, and pathogen levels) observed across the water treatment contexts such as drinking water and wastewater directly impact required oxidant dose.

While the operating principle of electrochemical disinfection through *in situ* generation of oxidants does not vary, the specific oxidant generated can impact the required dose across treatment contexts and introduce chemical reaction pathways that compete with pathogen inactivation.^28,62−65^ Likewise, the water composition across treatment contexts, and subsequently the oxidant demand for disinfection, can vary substantially (Figure 1).^11,12,32,66^ For electrochemical disinfection, the three treatment contexts evaluated in this study are water treatment, centralized wastewater treatment, and distributed wastewater treatment. Electrochemical disinfection in the water treatment context would occur after coagulation/flocculation, sedimentation, and filtration stages where relevant, analogous to traditional chemical disinfection processes.^67^ While the scale of centralized water treatment systems can vary, the composition and quality of the water to be treated is dependent on source and season. Within the centralized wastewater treatment context, electrochemical disinfection would likewise proceed similarly to traditional chemical disinfection processes after primary treatment and secondary biological treatment at a centralized treatment plant. In this study, electrochemical disinfection within the distributed wastewater treatment context occurs after waste dilution with flush water and other gray water sources, but independent of any physical, chemical, or biological pretreatment. This context is analogous to both emerging sanitation systems and more common septic systems used in resource limited settings worldwide.^68−70^

### 2.1 Water Composition

Similar to conventional disinfection systems, electrochemical disinfectant demand and system performance are strongly connected to water quality components, most significantly carbonaceous chemical oxygen demand (cCOD) and ammonia (Table 1).^12,62,71^ cCOD is a measure of the carbonaceous, oxidizable pollutants in the treated water and serves as an easily quantifiable analogue of oxidizable organic carbon in the system.^32,66^ In electrochemical disinfection, the oxidant is generated at the surface of the electrode and then diffuses into the bulk solution where the majority of pathogen inactivation occurs.^41,72^ However, as electrochemically generated oxidants lack inherent selectivity toward pathogens, the cCOD present in solution can scavenge the oxidant and lower the effective disinfectant dose.^12,47^ Because cCOD concentration can exceed 1000 mg L^–1^ in wastewaters, organic carbon can often constitute the majority of oxidizable species in treated waters.^32,73,74^ In addition to high levels of carbonaceous oxidant scavengers, many treatment contexts also display high levels of ammonia.

**Table 1:** Water Quality Components and Concentration Ranges Across Treatment Contexts^a^

	water quality components		
treatment context	cCOD (mg L^–1^)	NH_4_^+^(mg L^–1^-N)	CI^–^ ((mg L^–1^)
drinking water	3–90	0	<100
centralized wastewater	25–76	0–20	30–100
distributed wastewater	250–1000	10–50	30–100

a Adapted from Metcalf et al.^32^ and Davis.^67^

Ammonia increases disinfectant demand in electrochemical disinfection systems through direct and indirect oxidation or complexation with Cl-based oxidants.^62,63,71,75^ In direct oxidation, ammonium in solution at high pH is oxidized to elemental nitrogen on the surface of the electrode (eq 1).
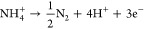
1

While direct oxidation of ammonia serves to denitrify the treated water, it also consumes electricity without producing oxidants, thereby reducing the effective dose at a given operating current and energy footprint of water treatment. Likewise, ammonium in solution can also be oxidized to nitrite (eq 2) and nitrate (eq 3).^62^
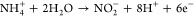
2
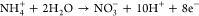
3

For noble metal electrodes, the direct oxidation of ammonia to elemental nitrogen is highly favorable over nitrogen oxyanions at lower electrode potentials (<0.0 vs SHE).^62,76^ For the more commonly used electrodes in electrochemical disinfection, such as dimensionally stable anodes (DSA) and boron-doped diamond (BDD) electrodes, between 11% and 65% of charge passed leads to nitrogen oxyanion production, with greater amounts occurring at more oxidative potentials.^75,77^ However, while nitrogen oxyanions may be produced at the anode depending on operating conditions, subsequent reduction at the cathode is also common.^78−80^

Under such conditions, produced nitrate and nitrite can be sequentially or directly denitrified at the cathode to elemental nitrogen or directly from nitrate to elemental nitrogen (eqs 4–6).
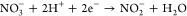
4
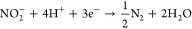
5
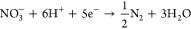
6

While accumulation of nitrate is possible, previous studies investigating both electrochemical disinfection and reduction of nitrate have shown near complete nitrate reduction to elemental nitrogen without significant reaccumulation of ammonia during operation.^78−80^ Ammonia can also reduce the effective disinfectant dose by scavenging the oxidant in the bulk solution similar to cCOD scavenging.^71,75^ Last, ammonia can react with electrochemically generated Cl_2_ to form chloramines (eqs 7–9) that reduce the strength of the oxidant.
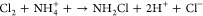
7
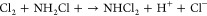
8
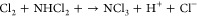
9

Because the chlorine produced through electro-chlorination is chemically identical to externally applied free chlorine additions, the electrogenerated chlorine can react with the initially produced monochloramine (eq 7) through subsequent reactions to form dichloramine (eq 8) and trichloramine (eq 8). The specific distribution of chloramine species is governed by solution features such as temperature, pH, and the chlorine to ammonia ratio as well as reaction time. As in chemical chlorination systems, electrochemical systems can be used for breakpoint chlorination, a process in which the chlorine is generated until all available ammonia has reacted and free chlorine is detected in the system.^78^ Due to the relative abundance of ammonia in electrochemical wastewater treatment contexts, chloramine should be present in any system generating free chlorine.^12,64,71,75,79^

### 2.2 Oxidant Types

**Figure 2:**
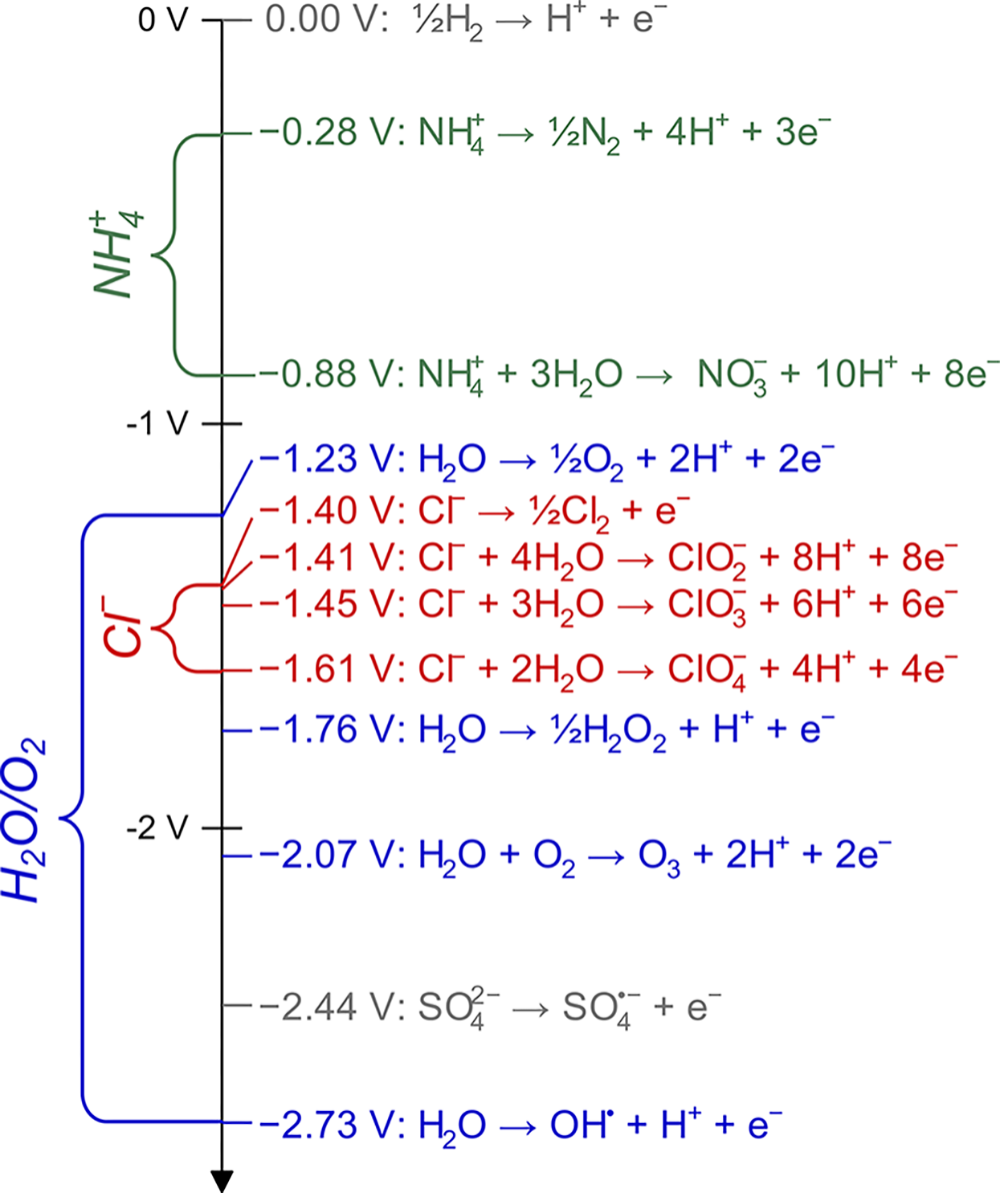
Standard oxidation potentials of important reactions for water disinfection including NH_4_^+^ (green), Cl^–^ (red), and H_2_O/O_2_ (blue) species groups. All oxidation potentials for nonradical species were calculated from Gibbs energies of formation under standard conditions. The oxidation potentials of SO_4_^•–^ and OH^•^ were taken from Armstrong et al.^82^

Multiple oxidants have been utilized for electrochemical disinfection by tuning the electrode type, water composition, and system operating voltage. Recent work has focused on generation of novel oxidants such as sulfate-based species (e.g., sulfate radicals, SO_4_^•–^, and S_2_O_8_^2–^) due to their high oxidative strength (Figure 2), wide range of operating pH, and longer lifetimes than typical radical species.^52,53,56^ However, while potentially promising for use in electrochemical disinfection systems, there is still insufficient disinfection and energetics data for sulfate-based systems to be included in this analysis. Accordingly, this work focuses on two of the most commonly employed oxidant types: free chlorine, Cl_2_, and hydroxyl radicals, OH^•^ (Figure 2). Each of these oxidants has distinct benefits and challenges for implementation in electrochemical disinfection systems.

Chlorine-based oxidants are the most widely utilized electrochemically generated disinfectants due to their relatively low oxidation potentials and the prevalence of chloride precursors in most waters of interest (Table 1).^12,60,61,81^ In chlorine-based systems, Cl_2_ is electrochemically generated on the electrode surface (eq 10) before diffusing into solution and hydrolyzing into both hydrochloric acid and the primary oxidant, hypochlorous acid (eq 11).
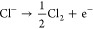
10
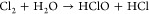
11

The standard oxidation potential of Cl_2_ is −1.40 V vs SHE, as compared to −2.44 and −2.73 V vs SHE for SO_4_^•–^ or OH^•^, respectively (Figure 2).^53−55,82^ Accordingly, the expected applied operating voltage and corresponding energy consumption of a disinfectant system utilizing Cl_2_ could be roughly 50% of a SO_4_^•–^ or OH^•^ based system. However, in order to electrochemically generate Cl_2_, the solution must contain Cl^–^ as a precursor (Figure 2). Because of the relatively high chloride concentration in urine and feces, most wastewater treatment contexts do not require any Cl^–^ supplementation.^73,83,84^ However, in most circumstances, the influent concentration of Cl^–^ in drinking water treatment is insufficient to produce Cl_2_ without an external source.^41,85^ Unlike hydroxyl radicals or other stronger oxidants, free chlorine can react with ammonia in solution to form chloramines as described above. Chloramines as disinfectants are commonly used to maintain a chlorine disinfectant residual in drinking water distribution systems due to their greater stability and lower associated disinfection byproducts (DBPs).^33^ However, both free chlorine and chloramines have generally higher required inactivation doses for most pathogens as compared to stronger oxidants such as OH^•^ and are sometimes ineffective in removing more recalcitrant pathogens such as *Giardia* or *Cryptosporidium* spp.^86−88^

Unlike free chlorine, hydroxyl radicals are the strongest commonly used electrochemically generated disinfectant but require the most energy to produce due to high oxidation potential (Figure 2).^55,65,89,90^ In addition to being one of the strongest oxidants, hydroxyl radicals are also some of the shortest lived oxidants.^33,91^ The combination of high oxidation potential and low lifetime (<1 μs) makes it difficult to measure the OH^•^ concentration in solution, particularly in the presence of other reactive oxygen species (ROS), and colorimetric assays and indicator compounds are often used to indirectly measure OH^•^ dose.^53,89,92,93^ While earlier studies questioned the relative impact of OH^•^ on pathogen inactivation due to this short lifetime, more recent studies have confirmed that hydroxyl radicals do directly contribute to inactivation, particularly for systems optimizing OH^•^ formation over other ROS.^12,55,91,93,94^ However, hydroxyl radicals are not suitable as a disinfectant residual during distribution in centralized treatment systems due to a short half-life. It is therefore critical to understand the solution composition and treatment context when evaluating the suitability and required disinfectant dose of electrochemical disinfection systems across oxidant types. While hydroxyl radicals do not as readily form trihalomethanes (THMs) and other DBPs commonly associated with chlorine-based disinfectants, they can form bromate and other toxic brominated compounds in bromide containing waters like other more common ROS disinfectants such as O_3_.^65,95−97^

## 3 Methods and Data Preparation

**Figure 3:**
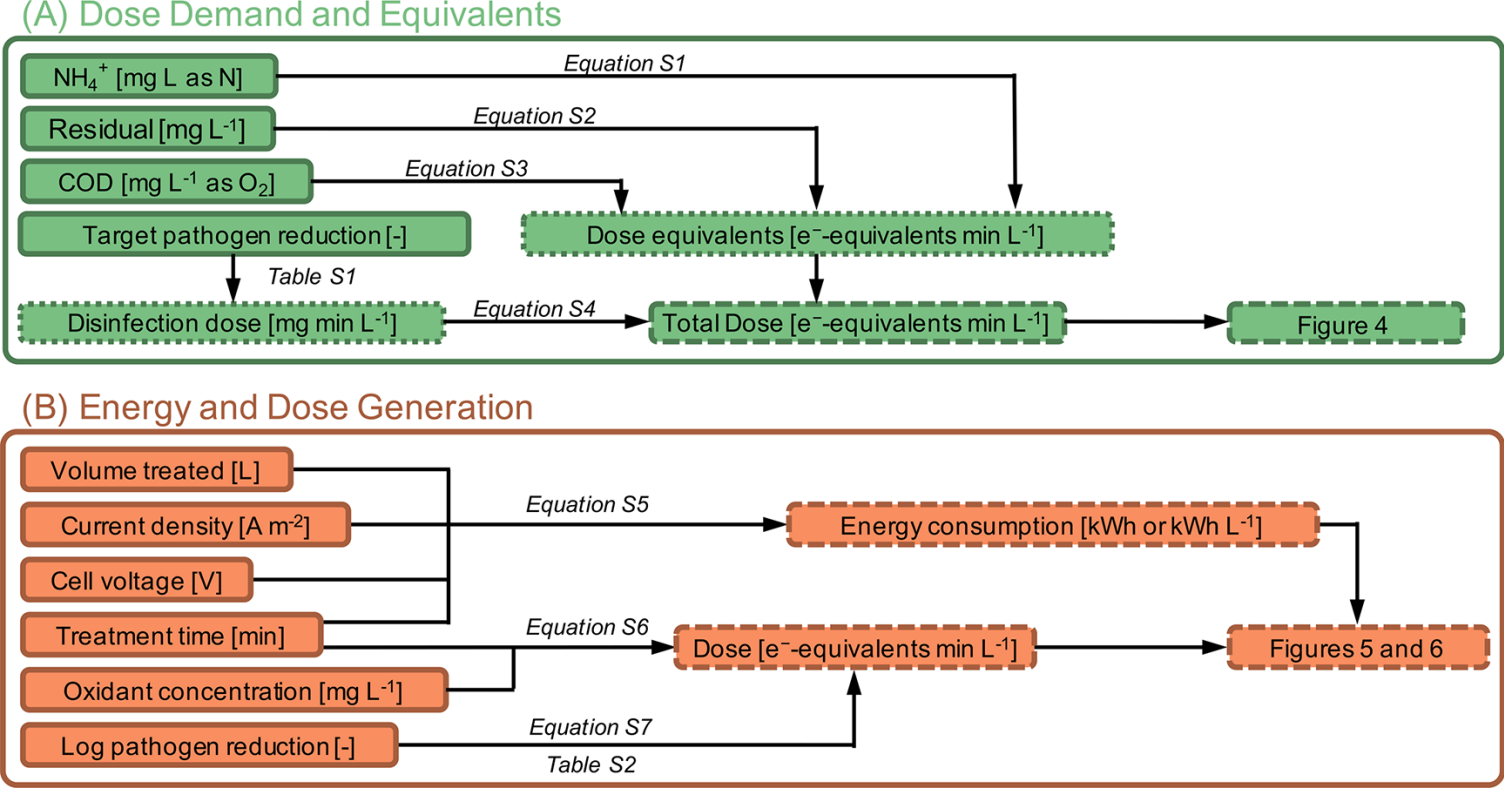
Flowchart showing the process of data acquisition and transformation utilized in this study. Transformation steps are further detailed in the SI.

A comprehensive literature review was conducted of studies reporting electrochemical oxidant generation. The scope of this survey was limited to oxidants for which oxidant-specific pathogen inactivation and energetic performance were previously reported or could be readily calculated; i.e., mixed oxidant systems such as broad ROS or the peroxone system were excluded. Accordingly, our analysis focused on free chlorine and hydroxyl radical systems. For inclusion within analyzed data, studies must have sufficiently reported oxidant generation parameters that enable estimation of disinfectant dose (Figure 3). Likewise, studies must have reported the energy consumption or cell operating characteristics to allow for the calculation of energy consumption. Given these requirements, only 16 of the 90 papers surveyed could include the analysis data set. The generated data set was used to analyze both the disinfectant dose demand for each oxidant across water treatment contexts and the corresponding energy of dose generation for each oxidant during operation.

### 3.1 Dose Demand and Equivalents

Typical ammonia concentrations, COD, target pathogen reduction, and disinfectant residual ranges across the three treatment contexts were used to calculate an effective total dose (mg min L^–1^ or e^–^-equivalents min L^–1^) for both free chlorine and hydroxyl radical systems (Figure 3A and Table S1). The free chlorine residual dose was calculated from common disinfectant residual concentration ranges with an assumed effective contact time of 1 min (see SIsection 1 for additional details on calculations).^32,67^ Because hydroxyl radical lifetimes are very short (<1 μs), the disinfection residual was assumed to be zero for those systems. Target pathogen reduction was set at 2-log inactivation of *Giardia* spp., which also corresponded to at least a 3-log inactivation of virus for all oxidants per U.S. EPA requirements.^33^ While Cl_2_ inactivation doses were drawn from U.S. EPA guidelines, required inactivation doses for OH^•^ have not be set by the U.S. EPA for *Giardia* spp. or viruses. Accordingly, estimated OH^•^ inactivation doses were calculated for these pathogens. Previously reported inactivation reaction rate constants of *E. coli* for both OH^•^ and O_3_ were used to determine the relative disinfection performance between the two oxidants.^91,93^ The primary mechanism of inactivation for O_3_ is the oxidative degradation of the outer membrane for bacteria and the destruction of the capsid or protein coating which protects DNA/RNA in viruses.^33^ Because similar mechanisms of bacterial and viral inactivation have been observed for OH^•^, estimated inactivation reaction rate constants of OH^•^ were calculated from those reported for O_3_ for viruses, *Giardia* spp., and *Cryptosporidium* spp.^33,91,93,95,98^

### 3.2 Energy and Dose Generation

Data from the literature which met the selection criteria were transformed to calculate system energy consumption (Wh or Wh L^–1^) and dose (mg min L^–1^ or e^–^-equivalents min L^–1^; Figure 3B). Dose calculation does not incorporate disinfectant decay over the reported contact time. For studies which did not report dose, disinfectant concentration, or contact time, dose was calculated using pathogen log inactivation assuming Chick–Watson inactivation kinetics (equation S7). For literature data, the whole cell voltage was used in calculation in order to accurately reflect actual energy consumption. When estimating minimum energy consumption for different water treatment contexts and operating conditions, the cell voltage was assumed to be identical to the anodic voltage (i.e., the cathode potential was assumed to remain at 0 V vs SHE). Counter electrode selection, solution composition (e.g., conductivity), electrochemical cell geometry, and operating conditions (e.g., hydraulic mixing) can vary substantially across systems and applications. Because of this high degree of uncertainty, these features were not incorporated into energy calculations for the simulated systems. Accordingly, simulated energy consumption values represent the minimum theoretical energy consumption under a given condition.

### 3.3 Electrochemical Process Modeling for Theoretical Energy Consumption

The minimum energy per dose (*E*_d_) for an electrochemical free chlorine disinfection system was calculated from using a simplified electrochemical process model (eqs 12–15)
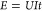
12
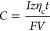
13
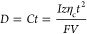
14
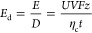
15where *U* is the voltage of the system (V), *I* is the applied current (A), *t* is the contact time (min), *C* is the concentration of Cl_2_ (mol L^–1^), *V* is the volume of the treated water (L), *F* is Faraday’s constant (C mol^–1^), η_c_ is the charge efficiency of Cl_2_ production, *D* is the corresponding disinfection dose (mol min L^–1^), and *z* is the number of electron equivalents per mole of oxidant (*z* = 2 for Cl_2_). In order to simplify the analysis, the voltage of the system was assumed to be identical to the anodic voltage. However, in reality, the cathodic voltage of the system will be nonzero, meaning that this analysis assumes a best-case scenario or minimal energy consumption. Likewise, charge efficiency was assumed to be constant with respect to cell voltage and contact time. In actual systems, the charge efficiency will vary with applied current or voltage and decreases to a steady-state value over time. With these simplifying assumptions, contact time and cell voltage are the primary operating parameters which can be altered to adjust performance.

## 4 Data Analysis

### 4.1 Impacts of Water Quality on Dose

**Figure 4:**
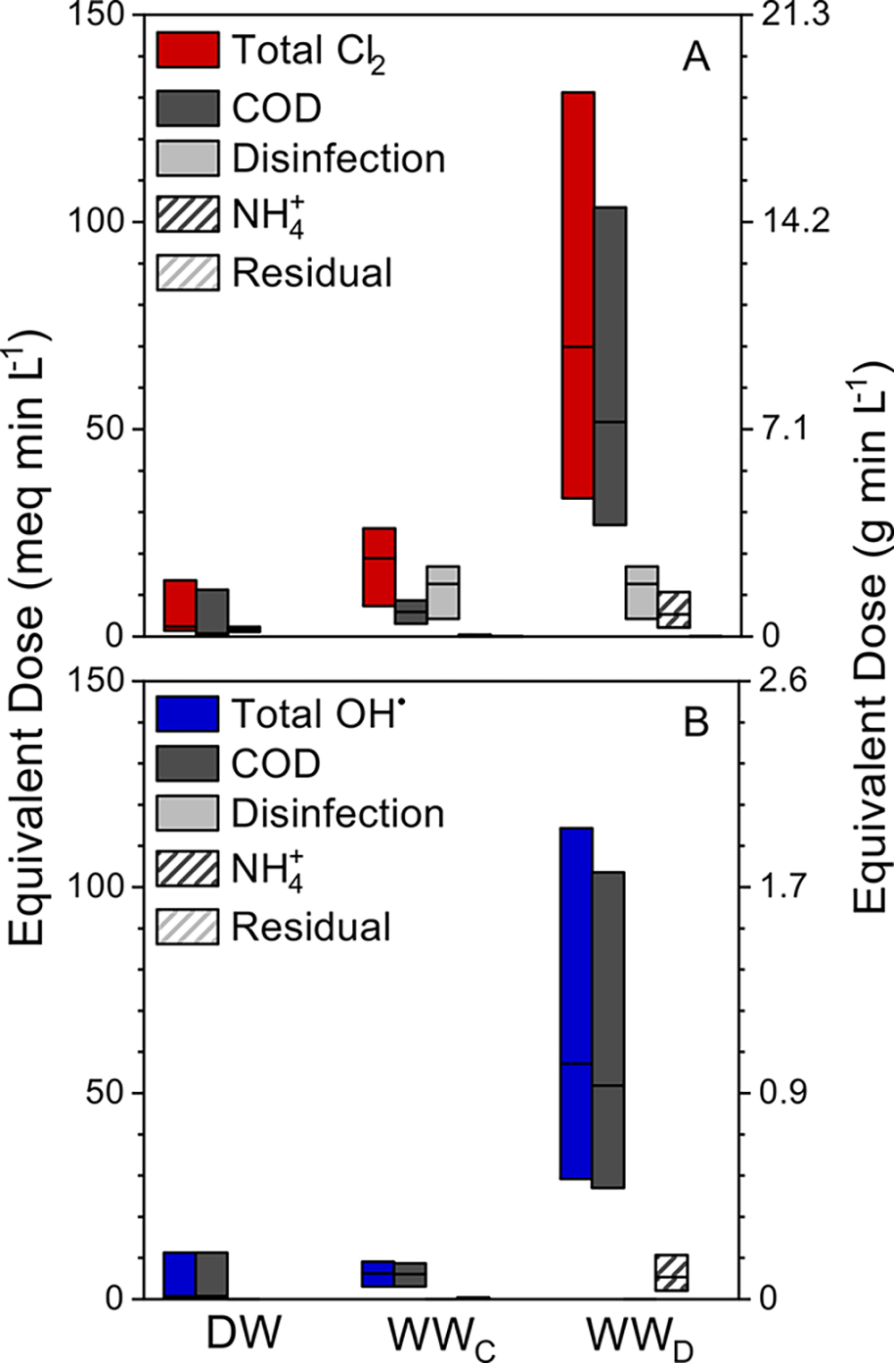
Effective oxidant dose necessary to treat typical drinking water (DW), centralized wastewater (WW_c_), and distributed wastewater (WW_d_) with Cl_2_ (A) and OH^•^ (B) as the primary oxidants. Bars show the range of demand due to water quality components with the median shown as a black line with total Cl_2_ and OH^•^ values corresponding to the sum of the four constituent components. To account for the difference in mass among the oxidants, effective dose is shown for electron equivalents (meq min L^–1^) on the primary axis with the more commonly reported mass dose (mg min L^–1^) shown on the secondary *y* axis.

For both oxidants, disinfectant demand across treatment contexts increases from drinking water to centralized wastewater to distributed wastewater treatment (Figure 4). The increased disinfectant demand is due to higher concentrations of cCOD, ammonia, and pathogens in both wastewater treatment contexts.^32^ Of each of these components, pathogen disinfection and cCOD are generally the most significant potential sources of oxidant consumption, accounting for an average of 53% and 44% of the oxidant demand across all treatment contexts and influent ranges, respectively (Figure 4). In the centralized wastewater treatment context, primary and secondary treatment reduce the cCOD and ammonia demand for an oxidant, which leads to pathogen inactivation accounting for the majority of the required effective dose.^32,46,99^ In this analysis, the distributed wastewater treatment context was assumed to have the same water quality conditions as centralized wastewater without treatment prior to disinfection (e.g., primary and secondary treatment). Without pretreatment, the oxidant demand for distributed wastewater treatment is substantially higher with cCOD and ammonia together accounting for at least 82% of total demand (Figure 4).

Across all treatment contexts, free chlorine based systems will require a greater expected dose than hydroxyl radicals (Figure 4). This difference in effective dose is due to the greater inactivation dose for Cl_2_ over OH^•^. For example, to achieve a 2-log inactivation of *E. coli*, the inactivation dose of Cl_2_ is approximately 3 orders of magnitude greater than the equivalent dose for OH^•^ (Figure 4A).^91,93^ Because of the relative importance of disinfection dose in free chlorine disinfection systems, treatment contexts which have higher pathogen levels (centralized and distributed wastewater) show the greatest disparity of required dose between Cl_2_- and OH^•^-based systems (Figure 4). Importantly, using mass-based dose metrics leads to significant apparent differences in required dose between the two oxidants evaluated. This effect is largely attributable to the difference in molecular weight of the respective compounds and not their relative disinfection efficacy (Figure 4). Therefore, we recommend using electron equivalents (meq min L^–1^) over more common mass-based metrics when reporting dose for electrochemical disinfection systems. For both oxidants, electrochemical systems are capable of the simultaneous transformation of cCOD, ammonia, and pathogens, however treating these additional water quality components will likely come at the expense of higher required dose and proportionally more energy consumption.

### 4.2 Experimental Energy Consumption for Dose Generation

The energy consumption in electrochemical disinfection is directly proportional to the operating voltage of the system (eq 12). During oxidant generation, the system anode potential (*E*_a_) can be approximated by the standard oxidation potential for the formation of the desired oxidant (Figure 2). The cathode is simultaneously polarized (*E*_c_), and the resulting potential difference across the electrodes determines the operating voltage of the system (*E*_a_ – *E*_c_). Depending on the configuration of the system, the cathodic potential can be of equivalent magnitude to the anodic potential, substantially increasing the energy consumption of the system beyond the thermodynamic mimimum.^48^ Likewise, while oxidant generation can theoretically occur at anodic potentials close to the standard oxidation potential, in practice, disinfection systems are typically operated at significant anodic overpotentials due to nonidealities associated with reactant concentration and electrode catalytic properties.^12,37,100^ Electrochemical systems can be operated under chronopotentiometeric (CP) or chronoamperometeric (CA) paradigms. In CA operation, the cell voltage can be increased beyond the standard oxidation potential, which correspondingly increases the operating current of the system and the rate of oxidant generation.^60^ Increasing cell voltage can also promote additional parasitic reactions, such as solution decomposition, thereby reducing the charge efficiency (the fraction of electrons used in oxidant generation). Conversely, in CP operation, a current is imposed on the system, and an associated overpotential is induced in the system. Due to the increased overpotential, higher operating currents are also associated with reduced charge efficiency and increased energy per mass of oxidant generated.^101^

**Figure 5:**
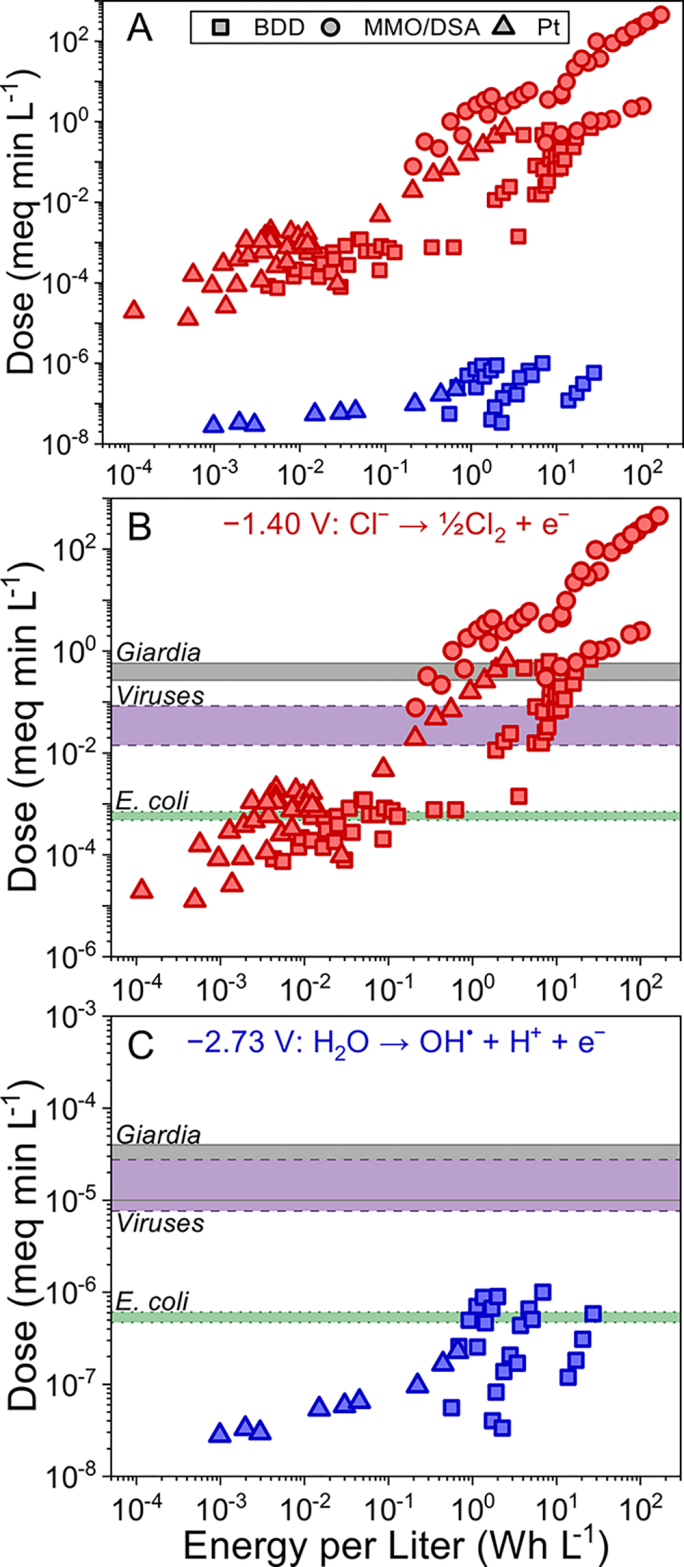
Dose generated per energy input for Cl_2_ (red) and OH^•^ (blue) as previously reported in the literature (A). Symbols indicate the electrode type utilized: boron-doped diamond (square), dimensionally stable anode/mixed metal oxide (circle), and platinum (triangle). Shade regions show estimated ranges of dose needed for 2 log inactivation of *E. coli* (green) and *Giardia* (gray) and 3 log reduction of viruses (purple) for Cl_2_ (B) and OH^•^ (C) as calculated per section 3.1.

Two of the most commonly reported electrode types for disinfection, dimensionally stable anode and boron-doped diamond, are used in part for their ability to operate at potentials within the water oxidation regime while still maintaining higher system charge efficiency (Figure 5). DSAs have been widely used in electrochemical disinfection and other chlorine generation applications.^12,48,75,101,102^ Electrochemical disinfection systems with DSAs can increase the practical operating voltage over standard electrodes (e.g., platinum) because of their high chlorine generation efficiency over water oxidation and other parasitic processes.^103^ Likewise, BDD electrodes have been widely used in electrochemical disinfection.^37,44,49,60,100^ Because BDD electrodes lack binding sites to facilitate water decomposition, they can be readily operated at higher cell voltages that are favorable for OH^•^ generation.^104^ However, operating at higher voltages corresponds to increased energy consumption as seen in reported systems using BDD electrodes (Figure 5). In addition to operating strategy and solution composition, both oxidant type and anode material can likewise influence the overall energy consumption of an electrochemical disinfection system.

In the studies sampled, free chlorine based systems generated up to 3 orders of magnitude more disinfectant dose than hydroxyl based systems when operated at comparable energy consumption (Figure 5). This difference in energy consumption per dose is partially due to the standard potential for generating each oxidant. The minimum cell voltage for free chlorine generation is 1.4 V vs SHE, whereas the minimum cell voltage for hydroxyl radical generation is 2.73 V vs SHE (Figure 5B and C). Of the free chlorine disinfection systems analyzed, those with BDD electrodes were typically operated at higher cell voltages (4–12 V) than those with DSA electrodes (3–5.5 V; Figure 5B). Most systems with DSAs produced a higher disinfection dose than those with BDD anodes at comparable energy expenditures, which is likely due to their higher chlorine generation charge efficiency.

While hydroxyl radicals are a stronger disinfectant than free chlorine, none of the OH^•^ based systems sampled from literature reported doses sufficient to achieve common inactivation targets for viruses or *Giardia* spp. per EPA surface water treatment rules requirements (Figure 5C). The maximum calculated dose for a hydroxyl based system was approximately 10^–6^ meq min L^–1^, which presents a substantial barrier for energy effective applicability given effective doses we estimate across treatment contexts (Figure 4B). For both free chlorine and hydroxyl radicals, dose generation is generally linearly proportional to energy consumption (Figure 5A). That suggests that hydroxyl radical systems would need to be operated in a highly energy intensive manner in order to achieve comparable doses to free chlorine systems. However, because of the difficulty in determining OH^•^ concentration, only two studies of OH^•^ based systems are included in this analysis. Likewise, challenges in quantifying OH^•^ dose and inactivation kinetics might lead to an underestimation of effective dose. While hydroxyl radical based systems have been used to inactivate bacterial pathogens, there have been no studies demonstrating electrochemically generated hydroxyl radicals as the sole oxidant for inactivating viruses or *Giardia* spp.^54,55,93,105^ Therefore, further studies are necessary to determine whether electrochemical systems can generate hydroxyl radical doses necessary to achieve virus or *Giardia* spp. inactivation. While doing so, researchers should also elucidate the relationship between energy consumption and dose generation for hydroxyl radical based disinfection systems across treatment contexts and operating strategies.

### 4.3 Impacts of Operating Conditions on Theoretical Energy Consumption for Dose Generation

**Figure 6:**
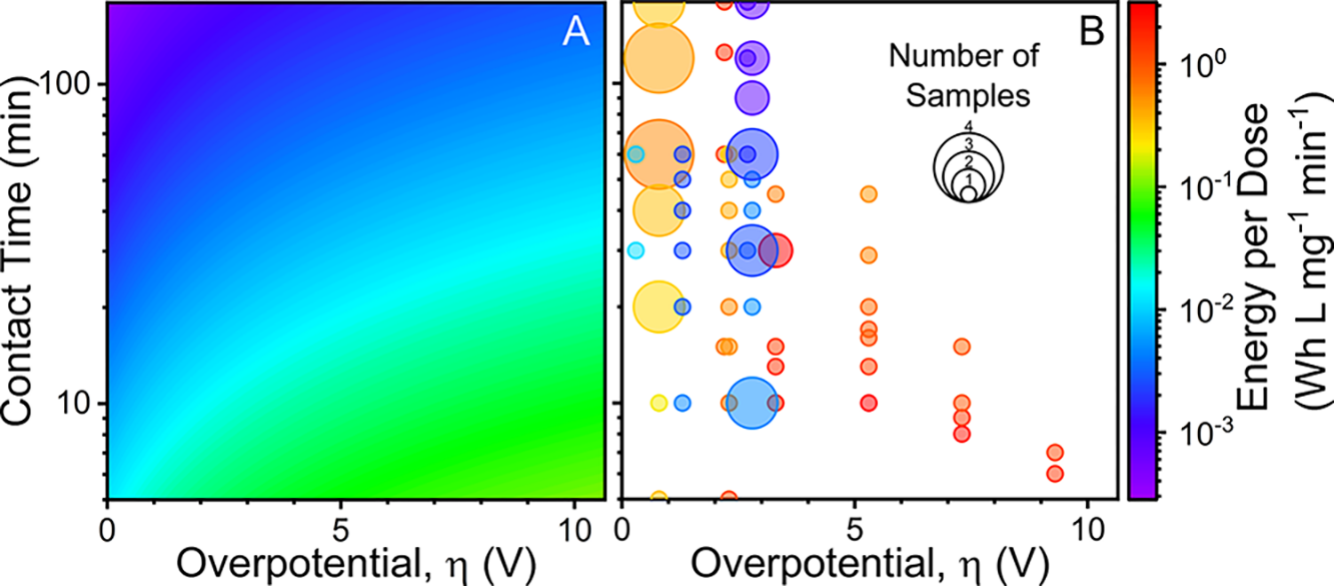
(A) Theoretical minimum energy per dose as a function of overpotential (horizontal axis) and contact time (vertical axis) for a simulated 1 L, Cl_2_ generating system. All cell voltage is assumed to be attributable to the working electrode. (B) Calculated energy per dose from Cl_2_ generating systems as reported in the literature plotted at corresponding overpotentials (horizontal axis) and contact times (vertical axis). Data reported for identical overpotential and contact times were averaged to a single energy per dose value with the bubble size indicating the number of samples averaged under those operating conditions.

With the ranges of contact time and overpotential typically reported in the literature, we utilized a simplified electrochemical disinfection process model to estimate the minimum theoretical energy consumption per dose for a free chlorine electrochemical disinfection system (Figure 6A). Simulated performance suggests that increasing contact time is a substantial means of reducing minimum energy per dose (Figure 6A). Because dose is proportional to the square of contact time (eq 10) while energy consumption is linearly proportional to contact time (eq 8), the energy per dose is inversely proportional to contact time at a given operating voltage. While following similar trends with respect to voltage and contact time, calculated energy per dose values for systems reported in the literature were 2 orders of magnitude greater on average than the estimated minimum under comparable conditions (Figure 6). This discrepancy is likely due, in part, to the nonideal charge efficiency experimentally observed versus the simulated ideal charge efficiency (i.e., η_c_ = 1; Figure S1).^80,84,101^ Although charge efficiency was not directly reported in many studies, some systems report performance near the theoretical minimum energy consumption (Figure 6B). This suggests that optimized operating conditions may be capable of achieving charge efficiency close to unity.

The much lower calculated system energy may also be due to additional system resistances and cathode overpotentials that were not included in the simplified model. In electrochemical systems, the conductivity of the solution treated can vary orders of magnitude depending on the total dissolved solids, from seawater ballast to relatively deionized drinking water.^40,51^ As the ionic conductivity decreases, the associated solution resistance of the system increases, which in turn leads to lower electrical current when operated at a fixed voltage and correspondingly lower oxidant generation.^106,107^ For example, the energy consumption of an electrochemical wastewater oxidation cell decreased by 88% when the solution conductivity was increased from 1.42 mS cm^–1^ to approximately 8 mS cm^–1^.^108^ This observation corresponds to previous recommendations which suggest that electrochemical disinfection systems are best leveraged in the contexts that have moderate to high existing conductivity (e.g., wastewater, seawater), can be easily supplemented with supporting electrolytes such as NaCl or Na_2_SO_4_, or treat water which undergoes an initial concentration such as membrane filtration concentrate.^47,51,60,107,108^

While a majority of experimental systems were operating at overpotentials below 3 V, the contact time was more varied across cell voltages (Figure 6B). The variance in contact time can be attributed in part to the different operational strategies and size of the experimental systems studied. While most of the electrochemical disinfection systems were operated in batch mode, wherein a single volume is treated for the duration of the disinfection process,^46,48,109,110^ some were capable of continuous operation, in which a treatment steam flows through the electrochemical disinfection reactor.^49,111^ Additionally, electrochemical treatment systems have been operated using an intermediate, semibatch system configuration, in which a batch reactor has reagent (such as NaCl, H_2_O_2_, or O_3_) periodically added.^112,113^ While continuous systems are capable of operating at long contact times using either very large systems or very low flow rates, batch and semibatch systems are more commonly used for distributed treatment due their typically smaller size and reduced complexity.^111^

Generally, systems operated at higher overpotentials consumed more energy per dose and were likewise operated at shorter contact times (Figure 6B). The relationship between contact time and cell voltage likely corresponds to attempts to reduce overall energy consumption at high overpotentials by reducing operating time. However, experimental data broadly confirm the expected performance trends from calculations with energy per dose decreasing with increasing contact time at a given cell voltage. The disparity between energy per dose at otherwise comparable operating conditions is likely due to differences in charge efficiency across experimental studies and conditions (Figure 6B). Additionally, the simplifying assumptions made in dose and energy calculation using extracted data from experimental studies may lead to an underestimation of energy per dose.

## 5 Outlook

Recent research aimed at increasing sanitation access worldwide and improving existing sanitation system resiliency has led to renewed interest in electrochemical disinfection systems for water treatment and disinfection. However, while numerous studies have investigated electrochemical disinfection system performance, reporting metrics have varied widely. When evaluating electrochemical disinfection systems both internally (e.g., across oxidant types, operating conditions, and system configurations) and externally (e.g., traditional chemical disinfection, filtration technologies, and thermal inactivation), it is critical to quantify both the disinfection efficacy and the energy consumed during operation.^114−117^ Therefore, we recommend that future work with electrochemical treatment should directly report both the dose generated and the energy consumption during generation to allow for comparison across operating conditions and strategies. If dose cannot be directly measured, as in the case of some OH^•^ based systems, then the concentration of oxidant should be estimated and reported using commonly accepted practices. Likewise, the charge efficiency of oxidant generation should be reported where possible in order to compare operating characteristics across the range of treatment contexts.

Hydroxyl radicals are a promising oxidant for use in electrochemical treatment due to their high oxidant strength and potential for inactivating highly recalcitrant pathogens such as *Cryptosporidium* spp.; however, their performance has not been as readily quantified in contrast to more common oxidants such as free chlorine.^31−33,87−90^ Therefore, additional studies are needed to both characterize the performance and navigate trade-offs between energy consumption and oxidant strength in hydroxyl radical based systems, particularly at sufficient doses for disinfection of viruses and *Giardia*. With an increased understanding of disinfection performance and energetics in OH^•^ based systems, the relative strengths and weaknesses of different oxidants can be identified for applicability across treatment contexts. Within resource limited settings, incorporating renewable energy sources such as photovoltaic cells presents an important pathway for sustainable electrochemical disinfection as similarly employed for other electrochemical processes.^118−121^

Last, alternative configurations and operational strategies should be investigated specifically targeting energy per dose reduction. Both experimental studies and simulated performance suggest that applying lower cell voltages over extended contact times leads to substantially reduced energy per dose (Figure 6). In both centralized and distributed treatment contexts, this will likely require engineering vessels which maximize residence time during disinfection. Additional studies are necessary to determine and navigate expected trade-offs between performance and economic viability of batch, semibatch, and continuous operation when utilized at high contact times. This is particularly true for larger, centralized systems which typically operate at very high throughput and may require continuous treatment systems. While such changes may represent an impractical barrier for existing centralized treatment systems, extended residence times are common in the batch or semibatch treatment systems widely utilized for distributed or semicentralized treatment in resource limited settings.^122,123^ Substantial challenges remain for the widespread deployment of robust electrochemical disinfection systems. However, when used within treatment contexts that promote intrinsic benefits, electrochemical disinfection shows substantial promise for improving global access sanitation and safe drinking water (Table 2).

**Table 2:** Benefits and Challenges of Electrochemical Disinfection Across Oxidant Types and Treatment Contexts^a^

	benefits	challenges
oxidants		
CI_2_	CI^–^ is among the most abundant anions in most waters; lower standard potentials than most electrochemically generated oxidants; longer lifetimes allow for disinfection residual; can combine with ammonia to form chloramines with lower DBPs and more stable residual	CI^–^ must be present in sufficient concentration or externally supplied; many oxidized CI species are toxic (CIO_2_^–^, CIO_3_^–^, etc.); current efficiency is dependent on CI^–^ concentration, limiting performance in low salinity waters; can generate chlorinated DBP such as trihalomethanes and haloacetic acids
OH·	strongest disinfectant among common electrochemically generated oxidants; can be generated without specific precursor ions (e.g., cl^–^); can also oxidize TOC and many micropollutants; capable of treating *cryptosporidium* at practical doses	highest standard potential of common electrochemically generated oxidants; radical species have very low lifetimes in solution; can generate BrO_4_^–^ and brominated compounds in Br^–^ containing waters
treatment context		
DW	low organic carbon levels reduce scavenging and necessary dose, depending on placement in treatment train; does not require onsite hazardous chemical storage; lower overall dose required compared to wastewater	May require CI^–^ addition if CI oxidant species are desired; most oxidants produced electrochemically have shorter lifetime, making residual generation difficult
WWW_c_	CI^–^ concentration is usually sufficient to generate CI oxidant; high NH_4_^+^ levels can scavenge more oxidized CI species (CIO_2_^–^, CIO_3_^–^, etc.) and produce chloramine; dose can be dynamically adjusted during severe events	high cCOD levels introduce competition for oxidant; electrochemical systems typically scale linearly for cost and energy demand
WWD_d_	electrochemical systems can be easily scaled down for portable units; a single unit can be used to electrochemically generate oxidants for simultaneous treatment of cCOD, NH_4_^+^, and pathogens	higher energy inputs are required for treatment of multiple target species; electrochemical systems typically have high capital cost compared to other methods

a DW, drinking water; WW_C_, centralized wastewater; and WW_d_, distributed wastewater.

## Supporting Information Available

Data transformation and calculation methods, impacts of contact time and charge efficiency on energy per dose, water composition ranges for dose demand calculations, disinfection kinetics parameters for dose calculations, and electron equivalents for water components and oxidants (PDF)
