# Supplementary material for: Electrochemical Disinfection in Water and Wastewater Treatment: Identifying Impacts of Water Quality and Operating Conditions on Performance
Source: Environ Sci Technol. 2021 Feb 22;55(6):3470–82. doi: 10.1021/acs.est.0c06254 (PMC7970539; doi:10.1021/acs.est.0c06254)
Supplement: Supplementary file 1 — es0c06254_si_001.pdf [file es0c06254_si_001.pdf]

Supporting Information: "Electrochemical disinfection in water and wastewater treatment: Identifying impacts of water quality and operating conditions on performance"

Steven Hand and Roland D. Cusick*

Department of Civil and Environmental Engineering

University of Illinois at Urbana-Champaign, Urbana, IL 61801-2352

Pages: 9

Figures: 1

Tables: 3

Section 1 – Data treatment and calculations

Dose demand and equivalents calculations

To compared disinfectant demand across treatment context and oxidant selection the equivalent dose demand (D_i) for each water quality component (i) was calculated based on the equivalents of electrons necessary to oxidize each component (Table S3). Ranges of typical ammonia, cCOD, and oxidant residual concentrations across the three treatment contexts were used with an assumed effective contact time of 1 min (i.e., 1 mg L^{-1} = 1 mg min L^{-1}) (Table S1). These mass-based doses then converted to equivalent dose for each species as follow:

$$D_{\mathrm{NH_4^+}} = \frac{C_{\mathrm{NH_4^+}}}{\mathrm{MW}_{\mathrm{NH_4^+}}} \cdot x_{\mathrm{NH_4^+}} \cdot 1\,\mathrm{min} \tag{S1}$$

$$D_{\mathrm{residual}} = \frac{C_{residual}}{MW_{residual}} \cdot x_{residual} \cdot 1\,\mathrm{min} \tag{S2}$$

$$D_{\mathrm{COD}} = \frac{C_{\mathrm{COD}}}{\mathrm{MW}_{\mathrm{COD}}} \cdot x_{\mathrm{COD}} \cdot 1\,\mathrm{min} \tag{S3}$$

where D_i is the equivalent dose of component i (e^--equivalents min L^{-1}), C_i is the concentration of component i (mg L^{-1}), MW_i is the molecular weight of component i, and x_i is the electron equivalents per mole of component or oxidant i. For MW_{COD} , the molecular weight of O_2 (32 g mol^{-1}) was used. the Mass-based dose demand for pathogen removal was calculated from typical ranges for the 2-log inactivation of *Giardia* spp (Table S1) and converted to equivalent dose as follows:

$$D_{\mathrm{disinfection}} = \frac{D_{\mathrm{m,disinfection}}}{\mathrm{MW}_{\mathrm{disinfection}}} \cdot x_{\mathrm{disinfection}} \tag{S4}$$

where $D_{m,i}$ is the mass-based dose of oxidant i. The total equivalent dose (D) was then calculate as the sum of the individual dose demands for each component.

$$D = D_{\mathrm{NH_4^+}} + D_{\mathrm{residual}} + D_{\mathrm{COD}} + D_{\mathrm{disinfection}} \tag{S4}$$

Energy and dose generation calculation

The energy consumed during oxidant generation in surveyed experiment studies was calculated from operation conditions as follows:

$$E = \frac{iAtU}{V\!\!\!/} \tag{S5}$$

where E is the energy consumed per liter (kWh L^{-1}), *i* is the operating current density (A m^{-2}), A is the electrode area (m^2), t is the time of operation (min), U is the operating cell voltage (V), and V̸ is the volume of water treated (L). The oxidant dose during operation was similarly calculated from operating conditions:

$$D = \frac{Ct}{MW_{ox}} \cdot x_{\mathrm{ox}} \tag{S6}$$

where D is the dose (e^--equivalents min L^{-1}) and C is the concentration of the oxidant (mg L^{-1}). To simplify our analysis, we did not account for oxidant decay during contact time. For those studies which did not report parameter necessary to calculation dose (dose, disinfectant concentration, or contact time), dose was calculated using pathogen log inactivation assuming Chick-Watson inactivation kinetics as follows:

$$\log \text{inactivation} = \log\left(\frac{C_X}{C_0}\right) = -k_{\mathrm{C-W}} C_D^n t \tag{S7}$$

where the log inactivation is the logarithm of the fraction of pathogens inactivated, $k_{C\text{-}W}$ is the coefficient of specific lethality (L mg^{-1} min^{-1}), t is the contact time (min), and n is the coefficient of dilution which as was assumed to be 1 for simplicity. Coefficients of specific lethality were calculated for *E. coli* for both free chlorine and hydroxyl radicals as well as for *Pseudomonas aeruginosa* and total colony-forming units (CFU) for free chlorine only (Table S2).

Studies included in surveyed dataset

(1) Cano, A.; Cañizares, P.; Barrera-Díaz, C.; Sáez, C.; Rodrigo, M. A. Use of Conductive-Diamond Electrochemical-Oxidation for the Disinfection of Several Actual Treated Wastewaters. Chem. Eng. J. 2012, 211–212, 463–469. https://doi.org/10.1016/j.cej.2012.09.071.

(2) Cho, K.; Qu, Y.; Kwon, D.; Zhang, H.; Cid, C. A.; Aryanfar, A.; Hoffmann, M. R. Effects of Anodic Potential and Chloride Ion on Overall Reactivity in Electrochemical Reactors Designed for Solar-Powered Wastewater Treatment. Environ. Sci. Technol. 2014, 48 (4), 2377–2384. https://doi.org/10.1021/es404137u.

(3) Bakheet, B.; Beardall, J.; Zhang, X.; McCarthy, D. What Is the Efficiency of Electro-Generation of Chlorine with a Solid Polymer Electrolyte Assembly? Chem. Eng. J. 2019, 364, 370–375. https://doi.org/10.1016/j.cej.2019.01.174.

(4) Raut, A. S.; Cunningham, G. B.; Parker, C. B.; Klem, E. J. D.; Stoner, B. R.; Deshusses, M. A.; Glass, J. T. Electrochemical Disinfection of Human Urine for Water-Free and Additive-Free Toilets Using Boron-Doped Diamond Electrodes. ECS Trans. 2013, 53 (17), 1–11. https://doi.org/10.1149/05317.0001ecst.

(5) Huang, X.; Qu, Y.; Cid, C. A.; Finke, C.; Hoffmann, M. R.; Lim, K.; Jiang, S. C. Electrochemical Disinfection of Toilet Wastewater Using Wastewater Electrolysis Cell. Water Res. 2016, 92, 164–172. https://doi.org/10.1016/j.watres.2016.01.040.

(6) Cho, K.; Kwon, D.; Hoffmann, M. R. Electrochemical Treatment of Human Waste Coupled with Molecular Hydrogen Production. RSC Adv. 2013, 4 (9), 4596–4608. https://doi.org/10.1039/C3RA46699J.

(7) Kerwick, M. I.; Reddy, S. M.; Chamberlain, A. H. L.; Holt, D. M. Electrochemical Disinfection, an Environmentally Acceptable Method of Drinking Water Disinfection? Electrochimica Acta 2005, 50 (25), 5270–5277. https://doi.org/10.1016/j.electacta.2005.02.074.

(8) Lacasa, E.; Tsolaki, E.; Sbokou, Z.; Rodrigo, M. A.; Mantzavinos, D.; Diamadopoulos, E. Electrochemical Disinfection of Simulated Ballast Water on Conductive Diamond Electrodes. Chem. Eng. J. 2013, 223, 516–523. https://doi.org/10.1016/j.cej.2013.03.003.

(9) Li X. Y.; Ding F.; Lo P. S. Y.; Sin S. H. P. Electrochemical Disinfection of Saline Wastewater Effluent. J. Environ. Eng. 2002, 128 (8), 697–704. https://doi.org/10.1061/(ASCE)0733-9372(2002)128:8(697).

(10) Nanayakkara, K. G. N.; Zheng, Y.-M.; Alam, A. K. M. K.; Zou, S.; Chen, J. P. Electrochemical Disinfection for Ballast Water Management: Technology Development and Risk Assessment. Mar. Pollut. Bull. 2011, 63 (5), 119–123. https://doi.org/10.1016/j.marpolbul.2011.03.003.

(11) Nadeeshani Nanayakkara, K. G.; Khorshed Alam, A. K. M.; Zheng, Y.-M.; Paul Chen, J. A Low-Energy Intensive Electrochemical System for the Eradication of Escherichia Coli from Ballast Water: Process Development, Disinfection Chemistry, and Kinetics Modeling. Mar. Pollut. Bull. 2012, 64 (6), 1238–1245. https://doi.org/10.1016/j.marpolbul.2012.01.018.

(12) Rajab, M.; Heim, C.; Letzel, T.; Drewes, J. E.; Helmreich, B. Electrochemical Disinfection Using Boron-Doped Diamond Electrode – The Synergetic Effects of in Situ Ozone and Free Chlorine Generation. Chemosphere 2015, 121, 47–53. https://doi.org/10.1016/j.chemosphere.2014.10.075.

(13) Raut, A. S.; Parker, C. B.; Klem, E. J. D.; Stoner, B. R.; Deshusses, M. A.; Glass, J. T. Reduction in Energy for Electrochemical Disinfection of E. Coli in Urine Simulant. J. Appl. Electrochem. 2019, 49 (5), 443–453. https://doi.org/10.1007/s10800-019-01292-4.

(14) Cano, A.; Barrera, C.; Cotillas, S.; Llanos, J.; Cañizares, P.; Rodrigo, M. A. Use of DiaCell Modules for the Electro-Disinfection of Secondary-Treated Wastewater with Diamond Anodes. Chem. Eng. J. 2016, 306, 433–440. https://doi.org/10.1016/j.cej.2016.07.090.

(15) Jeong, J.; Kim, J. Y.; Cho, M.; Choi, W.; Yoon, J. Inactivation of Escherichia Coli in the Electrochemical Disinfection Process Using a Pt Anode. Chemosphere 2007, 67 (4), 652–659. https://doi.org/10.1016/j.chemosphere.2006.11.035.
(16) Jeong, J.; Kim, J. Y.; Yoon, J. The Role of Reactive Oxygen Species in the Electrochemical Inactivation of Microorganisms. Environ. Sci. Tec*hnol.* **2006**, *40* (19), 6117–6122. https://doi.org/10.1021/es0604313.

Section 2 – Supplemental Figures

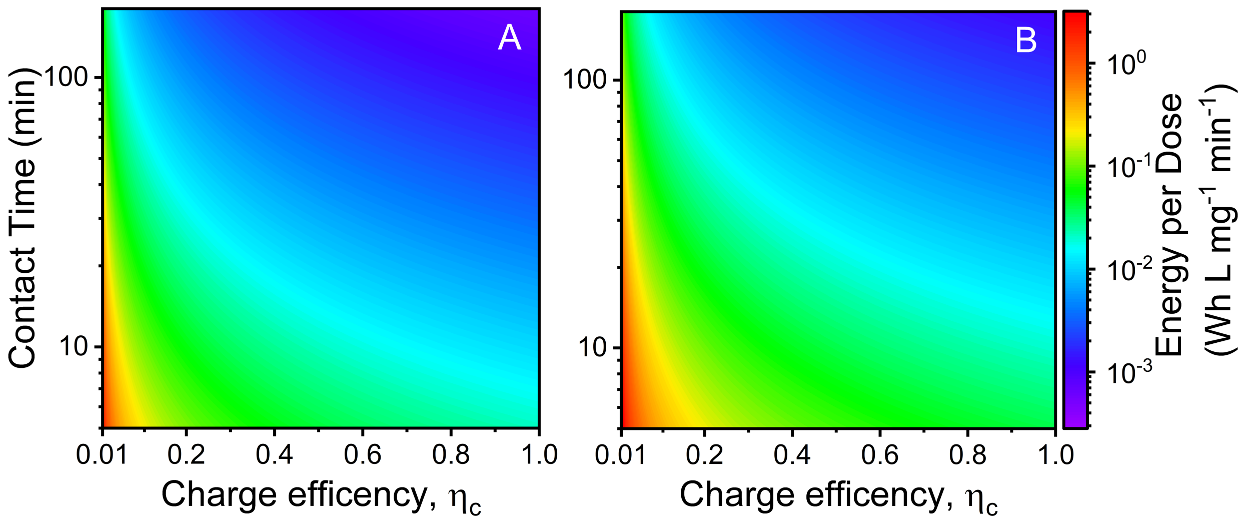

Figure S1. Theoretical minimum energy per dose as a function of charge efficiency and contact times for a simulated 1 L, Cl_2 generating system operated at overpotentials of 1 V (A) and 3V (B). All cell voltage is assumed to be attributable to the working electrode.

Section 3 – Supplemental Tables

Table S1. Water composition ranges for dose demand calculations.

treatment context	water quality components					
	cCOD (mg L^{-1})	NH_4^+ (mg-N L^{-1})	Disinfectant dose (mg min L^{-1})		Disinfectant residual (mg min L^{-1})	
			Cl_2	*$OH^\bullet$*	*Cl_2*	*$OH^\bullet$*
Drinking Water	3–90	0[a]	40–80	4–8 x 10^{-5}	0.1–0.3	0
Centralized Wastewater	25–76	0–20	150–600	1–2.5 x 10^{-3}	2–4	0
Distributed Wastewater	250–1,000	10–50	150–600	1–2.5 x 10^{-3}	2–4	0

[a]Drinking water treatment was assumed to have no ammonia present

Table S2. Chick-Watson parameters for dose calculations.

pathogen	coefficient of specific lethality, $k_{C–W}$	
	Cl_2	*$OH^{\bullet}$*
E. coli	50	12,500
P. aeruginosa	111	-
Total colony-forming units	0.02	-

Table S3. Electron equivalents for water components and oxidants.

Species	Electron equivalents (e^{-} $mole^{-1}$)	Corresponding Reactions
Ammonia	3	$NH_4^+ \rightarrow ½N_2 + 4H^+ + 3e^-$
cCOD	4	$2H_2O \rightarrow O_2 + 4H^+ + 4e^-$
Cl_2	2	$Cl^- \rightarrow Cl_2 + 2e^-$<br>$Cl_2 + H_2O \rightarrow HClO + HCl$
$OH^•$	1	$H_2O \rightarrow OH^· + H^+ + e^-$
